# Heart-derived endogenous stem cells

**DOI:** 10.1007/s11033-025-11001-4

**Published:** 2025-09-10

**Authors:** Keiko Inouye, Garrison White, Sadia Khan, Joseph Luba, Peyman Benharash, Finosh G. Thankam

**Affiliations:** 1 Department of Translational Research, College of Osteopathic Medicine of the Pacific, Western University of Health Sciences, Pomona, CA 91766-1854 USA; 2 Division of Cardiac Surgery, David Geffen School of Medicine at UCLA, Los Angeles, CA 90095 USA

**Keywords:** Cardiac-derived stromal cells, Cardiac regeneration, Myocardial infarction, Resident stem cells

## Abstract

Regenerative cardiology has emerged as a novel strategy to improve cardiac healing following ischemic injury. While stem-cell-mediated cardiac regeneration has garnered much attention as a promising strategy, its value remains debated owing to the lack of ideal stem cell source candidates. Resident/endogenous cardiac-derived stromal cells (CSCs) exhibit superior therapeutic potential due to their innate abilities to differentiate into cardiac cells, especially cardiomyocytes (CM). Emerging research has highlighted diverse endogenous CSCs phenotypes and sub-types as candidates for cardiac repair. Interestingly, CSCs promote healing through angiogenesis and regenerative paracrine signaling along with replenishing CM, and CM-like cells in the ischemic heart. Unfortunately, the clonogenic properties and translational potential of CSCs are minimally explored. This review examines the healing promise of a myriad CSCs such as c-kit + cardiac cells, Sca-1 + cells, cardiosphere-derived cells, side population cells, Bm1 + cells, cardiac atrial appendage cells, cardiac adipose cells, epicardial cells, and Isl1 + cells. Also, the review highlights the areas of improvement regarding the therapeutic applications of CSC to extrapolate into the clinical arena of cardiac management.

## Introduction

Heart disease is the leading cause of death among adults worldwide [1]. Advancements in pharmacotherapy, diagnosis and revascularization have improved patient outcomes. However, irreversible fibrosis following an acute ischemic event leads to chamber dilation, heart failure and arrythmias. Lack of sufficient myocardial blood flow during a myocardial infarction (MI) result in CM apoptosis/necrosis of CM’s with eventual fibrosis mediated primarily by myofibroblasts as an end product of healing. This collagenous scar has limited contractile properties, leading to the development and progression of heart failure [2]. Recently, the regenerative properties of stromal cells have been explored to prevent cardiac fibrosis. Stromal cells are currently utilized in clinical trials, and research continues to further define their regenerative applications. As of 2020, the WHO International Clinical Trials Registry has up to 3000 studies involved in stromal cells [3]. Notably, current research is pursuing human pluripotent stromal cells (hPSCs) as a potential therapeutic alternative to CSCs. The pioneering investigations by Takahashi & Yamanaka laid foundations for hPSC due to their ability to differentiate into any cell lineage [4]. However, hPSCs require extensive programming for differentiation.

While resident CSCs carry inherent cardiac regenerative potential [5–7], their isolation and expansion has proved challenging [8]. This hurdle has led to the pursuit of other stromal cell lines, such as hPSCs, for cardiac regeneration; however, CSCs hold the highest potential for cardiac regeneration owing to their cardiac lineage specificity [8]. This review discusses the current modalities and perspectives regarding the regenerative biology of CSCs and other cardiac stem cells.

### Stromal cell-mediated cardiac healing

**Fig. 1:**
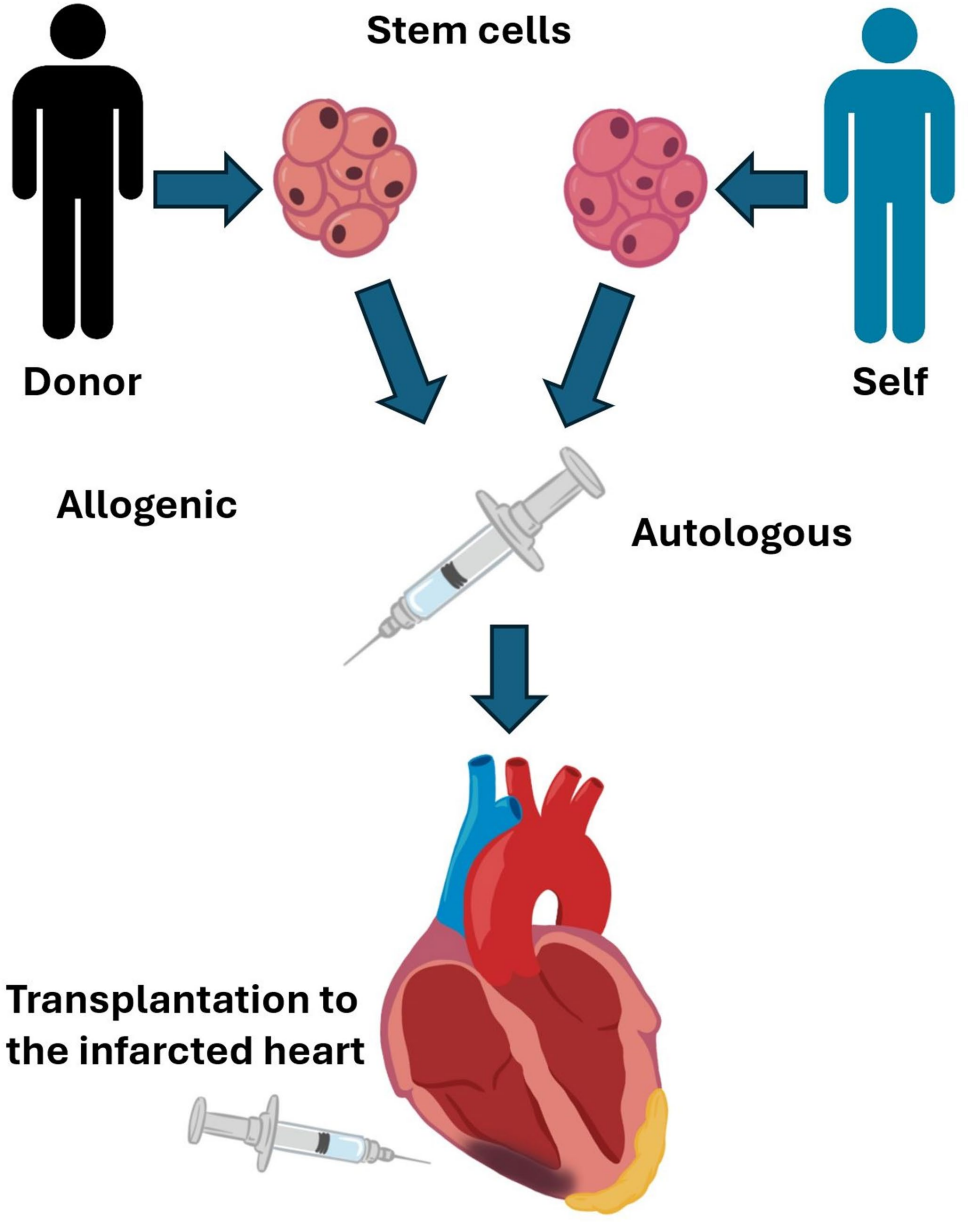
Methods of stem cell-mediated transplantation for cardiac healing

Given the limited regenerative capability of the human heart, stromal cell therapies may provide a replacement for fibrotic cardiac tissue following an acute MI. Notably, induced pluripotent stromal cells contribute to most current data regarding stromal cell-mediated CM regeneration. The stromal cells have been isolated and/or expanded from autologous or allogeneic sources for transplantation (Fig. 1). Additionally, skeletal myoblasts (another source of stromal cells) have been used in cardiac regenerative therapy due to their ability to differentiate into myotubes. Poor electric conductivity, limited integration with myocardium and incomplete differentiation into CM hinder the translational application of extra-cardiac stem cells [9]. Bone marrow-derived stromal cells are defined by their surface markers: hematopoietic bone marrow stromal cells (BM-HSCs) and bone marrow-derived mononuclear cells (BMMNCs) [10]. Apart from their ready availability and maintenance, BMMNCs specifically demonstrate strong paracrine effects and cardiac regenerative potential [9]. In early clinical trials, these cells demonstrated the capability of differentiating into CM; however, displayed inconsistent clinical outcomes [11].

Current research is focused on characterizing the efficacy of endogenous CSCs. Cardiosphere-derived cells are cardiac progenitor cells (CPCs) capable of differentiating into CM, endothelial cells, and smooth muscle cells in vivo as evident from the improved left ventricular function following acute MI in rodent models [12]. However, these cells have shown poor engraftment in preclinical trials, suggesting potential benefits to be mediated through paracrine action rather than intrinsic contractility [11].

**Fig. 2:**
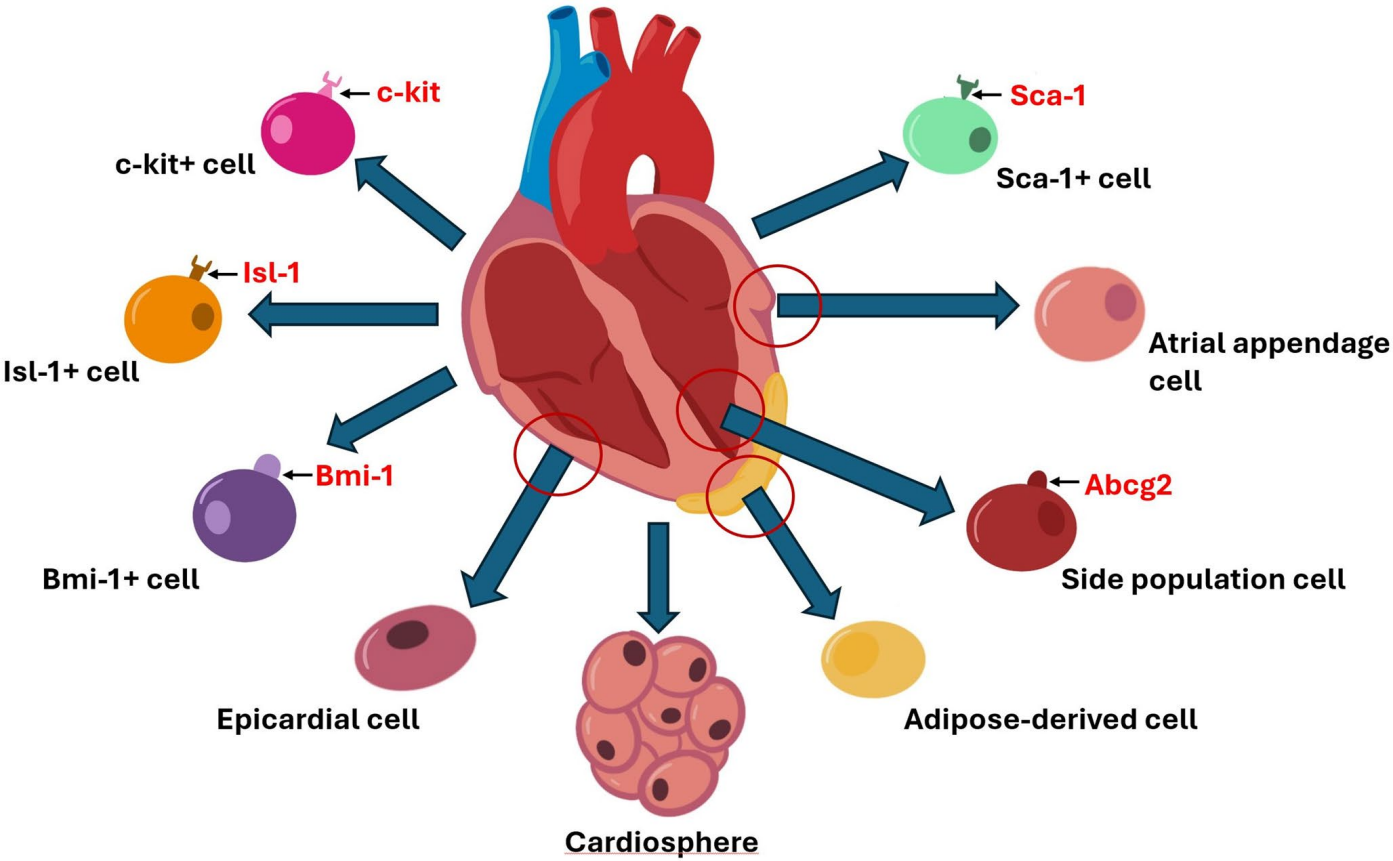
Overview of resident CSCs, surface receptors, and locations in the heart

Resident/endogenous CPCs are found in the atria, ventricles, epicardium, and pericardium [13]. Although the heart was thought to be a post-mitotic organ, several studies have demonstrated that resident CPCs can be activated following an injury [7, 14]. As these cells are autologous and pre-committed to the cardiovascular lineage, they offer therapeutic promise over extra-cardiac stromal cell populations. The regenerative capability of these cells has been demonstrated through isolation from the patient, amplification ex vivo, autologous administration, and via their endogenous activation at the injured cardiovascular tissue [15]. Hence, resident cardiac cells offer high therapeutic potential with minimal cost and ethical ramifications. The following section comprehends the regenerative qualities and pro-healing mechanisms underlying diverse CPCs. The key characteristics and biomarkers of CPCs are depicted in Table 1; Fig. 2.

### Endogenous cardiac stromal cells

#### C-kit + cells

Cell growth factor receptor KIT (c-kit) is a proto-oncogene known to play a vital role in the inflammatory response. c-Kit has been used to identify populations of CSCs with clonogenic/self-renewing properties [16]. Studies have shown that in response to hypoxic conditions, c-kit + cells activate the HIF-1α pathway and upregulate genes associated with inflammation and repair [17–19]. Overall, c-kit + CSCs aid in the healing process post-infarction, reducing hypertrophy and fibrosis, thereby improving cardiac function [17]. Notably, KIT is present in hematopoietic stromal cells, cardiac mast cells, postnatal CM, coronary endothelial cells, and epicardial cells [18, 20]. CM progenitor c-kit + cells are differentiated from other lineages based on NKX2.5, GATA4, and MEF2C expression and the absence of hematopoietic markers [18, 21]. These cells exhibit regenerative and clonogenic properties in vitro while exemplifying an affinity for differentiation into CM, endothelial cells, and smooth muscle cells, promising cardiac regeneration [18, 22]. Ellison et al. [19] showed that injection of c-kit + CSCs into infarcted myocardium induced CM differentiation [19, 22]. Other studies have also found that the presence of c-kit plays a variable but important role in the expansion of CM following an MI [18, 23, 24]. Importantly, c-kit + CSCs exhibit favorable electrophysiologic and contractile properties which are critical for cardiac regeneration [10].

Contrastingly, Aquilla et al. demonstrated that c-kit haploinsufficiency impairs cardiac stem cell growth, myogenic potential, and regenerative capacity [25]. C-kit haploinsufficient mice failed to demonstrate CSC activation and displayed decreased CM formation in response to isoproterenol overdose-induced cardiac dropout. Transplantation of wtCSCs restored the impaired regenerative cardiac phenotype observed in the c-kit haploinsufficient mice [25]. Furthermore, Vicinanza et al. (2017) reported that adult CSCs are multipotent and robustly myogenic, with c-kit expression being necessary, however not sufficient for their identification [26]. Also, CD45(-) c-kit + cell-derived clones, a subset representing only 1–2% of all c-kit positive cardiac cells, were capable of differentiation into functional and spontaneously contracting cells with a cellular, electrophysiological, and transcriptome phenotype characteristic of CMs when stimulated by Wnt inhibitors and TGF-β family agonists in vitro [26]. Furthermore, progeny derived from these clones demonstrated functional myocardial regeneration when injected into infarcted myocardium.

Notably, changing the view of c-kit as a marker displayed additional challenges. Earlier studies often treated c-kit expression as an identifier of a separate progenitor pool; however, more recent perspectives emphasize its dynamic nature. Zhou and Wu highlighted that c-kit expression in cardiac cells reflects an interplay between expression levels, fate, and function [27]. Given that c-kit is expressed in both cardiomyocytes and nonmyocytes, interpreting c-kit as a marker is trivial. Gude and Sussman further emphasized that c-kit expression changes with developmental stage and in response to injury as being upregulated in dedifferentiating adult mammalian CMs. Also, traditional lineage-tracing and knock-in models underestimate the role of c-kit + cells [28]. Moreover, single allele knock-in replacements often reduce overall c-kit expression, which may impair their regenerative function than the wild-type counterparts. In addition, transgenic reporter models often fail to detect dynamic or injury-induced c-kit expression [28]. Overall, these observations suggest that c-kit reflects context-dependent regenerative potential rather than a static marker of endogenous stem cells.

### Stromal cell antigen-1+ (Sca-1+) cells

Sca-1 + cells play a crucial role in inhibiting CM apoptosis, healing post-MI, and inducing stromal/progenitor cell differentiation into CM [29]. Sca-1 + cells from cardiac interstitial and vascular factions differentiate into CM using 5-azacytidine, upregulating the cardiac transcription factors such as GATA4, MEF2C, and TEF1 [18, 21]. Prior work has reported on the *de novo* generation of CM from Sca-1 + cells. Additionally, recent studies have shown a predominantly endothelial lineage rather than intrinsic cardiac precursor cells [30, 31]. These findings suggest that Sca-1 + cells play a role in the healing process due to hematopoietic properties and angiogenesis rather than the proliferation and growth of new CM [10, 30, 31]. Deleting the Sca-1 gene in vivo impaired cardiac function, likely due to a decrease in vascular proliferation suggesting their regenerative function by angiogenesis to restore the blood flow [18]. Differentiation of CM observed from Sca-1 + cells is due to signaling by sVCAM-1 secretion and its receptor VLA-4. This pathway promotes cardiomyogenesis through improved engraftment and promotes migration and renewal of both endogenous and transplanted Sca-1 + cells [32].

### Cardiosphere-derived cells (CDC)

CDCs are a heterogeneous cluster of CSCs expressing cardiac-specific factors, GATA4, MEF2C, and MKX2-5, surrounded by stromal cells [18, 21, 22, 33]. CDCs at the core propagate and have multilineage potential [10]. A seminal study by Smith et al. [34] demonstrated that co-culture of these cells with CM drove their differentiation into CM [34]. Additionally, the injection of CDCs into infarcted tissue increased the density of CM and vascular and endothelial cells signifying their paracrine mechanisms [18, 21, 22, 29, 33]. Exosomes isolated from CDCs increased angiogenesis, proliferation, and improved CM survival [33, 35]. Also, scar reduction was observed following injection of CDC, along with increased viable myocardium and improved function post-injury [10, 21]. A seminal study by Grigorian-Shamagian et al. [33] demonstrated that CDCs secrete exosomes that specifically influence telomerase activity in aging tissue suggesting their cardioprotective role [33]. Despite the observed regenerative effects, these cell-types fails to restore significant cardiac repair and/or function. A seminal study by Zhao et al. showed that mice injected with CDCs failed to display increased cardiac function compared to a control group that received saline [36, 37]. Additionally, in the ALLSTAR study, the patients treated with CDCs showed minimal reduction in scar size and similar ventricular function after 6 months compared to placebo group concerning the efficacy of CDCs in cardiac regeneration [38].

### Side population cells

Cardiac side population cells (cSPCs) are tissue-specific hematopoietic cells that differentiate into CM, endothelial cells, fibroblasts, and smooth muscle cells [18, 39, 40]. cSPCs express high levels of Sca-1 and have been identified in bone marrow and cardiac tissue [41]. Cells originating from cardiac tissue express cardiac-specific factors NKX2-5, MEF2C, and GATA4, as well as surface marker Abcg2+ (also known as Bcrp1) [40]. Abcg2 is expressed in both stromal and tumor cells and allows Abcg2-CreER to promote cell-cycle reentry and proliferation, thus influencing their regenerative properties [42] cSPCs have shown to differentiate into CM via co-culture with CM or alone [39, 40].

### Bmi1 + cardiac stromal cells

B cell-specific Moloney murine leukemia virus integration site 1 (BMI1) is a transcriptional suppressor gene involved in self-renewal and proliferation of tissue [22, 43, 44]. BMI1is highly expressed in cancers and heart tissue during injury, ischemia, and oxidative stress [44]. Bmi1 promotes the growth and proliferation of CSCs and has been studied as a potential therapeutic candidate. Bmi-1 + CSCs differentiate into CM-like cells, vascular endothelial cells, and smooth muscle-like cells [22, 44, 45]. Deficiency of Bmi-1 + cells impairs angiogenesis and progression to cardiomyopathy, suggesting their potential role in cardiac healing [44]. These cells support revascularization and remodeling via *de novo* angiogenesis and CM after MI [46, 47]. Under hypoxic conditions, the HIF-1α-activated IL-22/IL-22R1 pathway upregulates Bmi1, which in turn modulates the self-renewal and proliferation of endogenous CSCs. This signaling cascade is essential for cardiac repair and regeneration following acute myocardial ischemia [48]. However, Bmi1 has been shown to promote cardiac fibrosis and dysfunction post-MI by inhibiting PTEN and activating the PI3K/Akt/mTOR pathway [49]. These pathways underscore the role of Bmi-1 + cardiac stromal cells in modulating cardiac repair mechanisms and maintaining cardiac homeostasis.

### Cardiac atrial appendage stromal cells

Cardiac atrial appendage stromal cells (CASCs) are, as their name suggests, present in the atrial appendages and are phenotypically unique from other CSCs. CASCs exhibit high aldehyde dehydrogenase activity, protecting these cells against cytotoxicity and hypoxic stress and making them prime candidates for regenerative therapies [10, 20, 50]. CASCs have been shown to promote angiogenesis through secretion of various growth factors, including vascular endothelial growth factor (VEGF), endothelin-1 (ET-1), and insulin-like growth factor binding protein (IGFBP-3) which enhance endothelial cell proliferation, migration, and tube formation [51]. In vivo studies have demonstrated that CASCs engraft and differentiate into functional cardiomyocytes when transplanted into MI models, leading to improved cardiac function and reduced adverse remodeling [52]. Additionally, CASCs were shown to reduce fibrotic scar tissue and improve left ventricular function when transplanted into MI models [53]. Koninckx et al. [20] demonstrated that isolated CASCs were more reliable and reproducible than CSCs, suggesting their superior regenerative potential [20]. Evens et al. [50] also revealed the cardioprotective effects of CASCs against advanced glycation end-products (AGEs) by the activation of the receptor for AGEs [50]. Furthermore, CASCs demonstrate potent immunomodulatory properties. These cells exhibit low immunogenicity by lacking co-stimulatory molecules and expressing inhibitory ligands such as PD-L1 and PD-L2, inhibit T cell proliferation through factors including indoleamine 2,3-dioxygenase (IDO), and secrete anti-inflammatory cytokines IL-10 [54, 55]. Moreover, CASCs induce regulatory T cell (Treg), evidenced by the increased FoxP3 + CD4 + CD25(high+) cell populations, further supporting their potential for allogeneic cell therapies [56].

### Cardiac adipose cells

Cardiac adipose-derived stromal cells (CADSCs) are a specific population of progenitor cells isolated from the adipose tissue surrounding the heart, particularly the epicardial fat. These cells exhibit characteristics similar to mesenchymal stem cells, including the expression of markers such as CD105, CD44, CD90, and CD29 [57]. CADSCs derived from the epicardium expressing cardiac-specific biomarkers (MEF2C, NKX2-5, TBX5, IRX4), have a strong propensity to differentiate into CM [58, 59]. They represent promising candidates for regenerative therapy due to their differentiation into diverse cardiovascular cell types, including endothelial cells, vascular smooth muscle cells, and beating CM [60–63]. CADSCs were shown to secrete factors that protect myocardial cells from apoptosis, inflammation, and fibrosis [63]. CADSCs post-MI show an upregulation of ribosomal proteins RPL10A, RPL14, RPL30, RPS18, FAU-40 (RPS30), and RPSA (Laminin Receptor, LR) which modulate immune response and aid in healing [64, 65]. Other studies by Cha et al. [66] showed that epicardial adipose tissue expresses increased levels of CRSP2, HSP27, IL-8, and downregulation of Cofilin-1 and HSP90 under ischemic conditions, which modulate inflammation and healing [66, 67]. Preclinical studies have demonstrated that CASCs improve cardiac function and reduce infarct size when transplanted into rodent models of MI [57]. In models of chronic ischemic cardiomyopathy, CASCs were shown to upregulate genes associated with angiogenesis and monocyte/macrophage differentiation, further indicating their role in long-term cardiac repair and remodeling [68].

### Epicardial cells (EpC)

Epicardial cells (EpC) represent the outermost layer of the heart and give rise to smooth muscle, endothelial cells, and stromal cells [18]. EpC are heterogeneous, including KIT + and other progenitor cells that express pro-angiogenic and healing properties [18, 65, 69]. EpCs use paracrine signaling following injury by secreting factors such as adenosine, tenascin-C (TNC), and hepatocyte growth factor (HGF)/IgG complexes, which modulate inflammation, promote angiogenesis, and protect vascular integrity, thereby contributing to cardiac repair and regeneration [70, 71]. Transplantation of EpCs in vivo showed improved cardiac repair and function due to increased VEGF-A, FGF2, and PDGF-C signaling [21]. Human embryonic stromal cell-derived EpCs promoted healing and regeneration via paracrine mechanisms [69, 72]. Additionally, EpCs suppress inflammation and promote reparative macrophage polarization by secreting intelectin 1 (ITLN1), which interacts with IFN-β, and enhance vascular protection through HGF/IgG complexes that activate the RYK receptor [73]. Furthermore, human EpCs release extracellular vesicles containing miRNAs including miR-30a, miR-100, miR-27a, and miR-30e, which promote CM proliferation and improve cardiac function [74]. Wasserman et al. [69] showed that oxytocin signaling plays an essential role in EpC proliferation and regeneration following injury, thus providing a mechanism for producing EpCs from precursors and increasing the activity of human-induced pluripotent stromal cell-derived EpCs [69].

### Isl-1 + cells

Isl-1 is expressed in cardiac mesodermal progenitor cells during development in the first and second heart fields and cardiac neural crest cells [13, 18, 75]. Mesodermal progenitor cells contribute to CM, endothelial cells, and smooth muscle cells during the development of the heart, and Isl-1 + cells with cardiac markers, such as NKX2-5, have been shown to differentiate into CM and smooth muscle cells in vitro [18]. Cohen et al. [75] reported that these cells formed new cardiac tissue in response to Wnt/beta-catenin signaling [75]. Xiang et al. [76] also showed significant improvement in cardiac function post-injury in a rat model with the addition of cells over-expressing Isl-1 [76]. Bartulos et al. [77] showed that Isl-1 + CPCs, when transplanted into murine hearts post-MI, differentiated into cardiomyocytes and endothelial cells, integrated into the myocardium, and promoted angiogenesis demonstrating a significant reduction in the infarct size and improved left ventricular function (70). Similarly, Ye et al. [78] found that Isl-1-expressing cells derived from cardiospheres differentiate into various cardiac cell types and improve cardiac function by promoting angiogenesis and reducing fibrosis in post-MI hearts [8, 78]. Despite these advances, the precise role of Isl-1 + cells in adult heart regeneration remains unclear. Some studies suggest limited direct involvement in post-injury repair while some highlight the potential of Isl-1-derived progenitors for cell therapy and the beneficial effects of Isl-1 gene or VEGF mRNA delivery on cardiac function and vascularization [18].

**Fig. 3:**
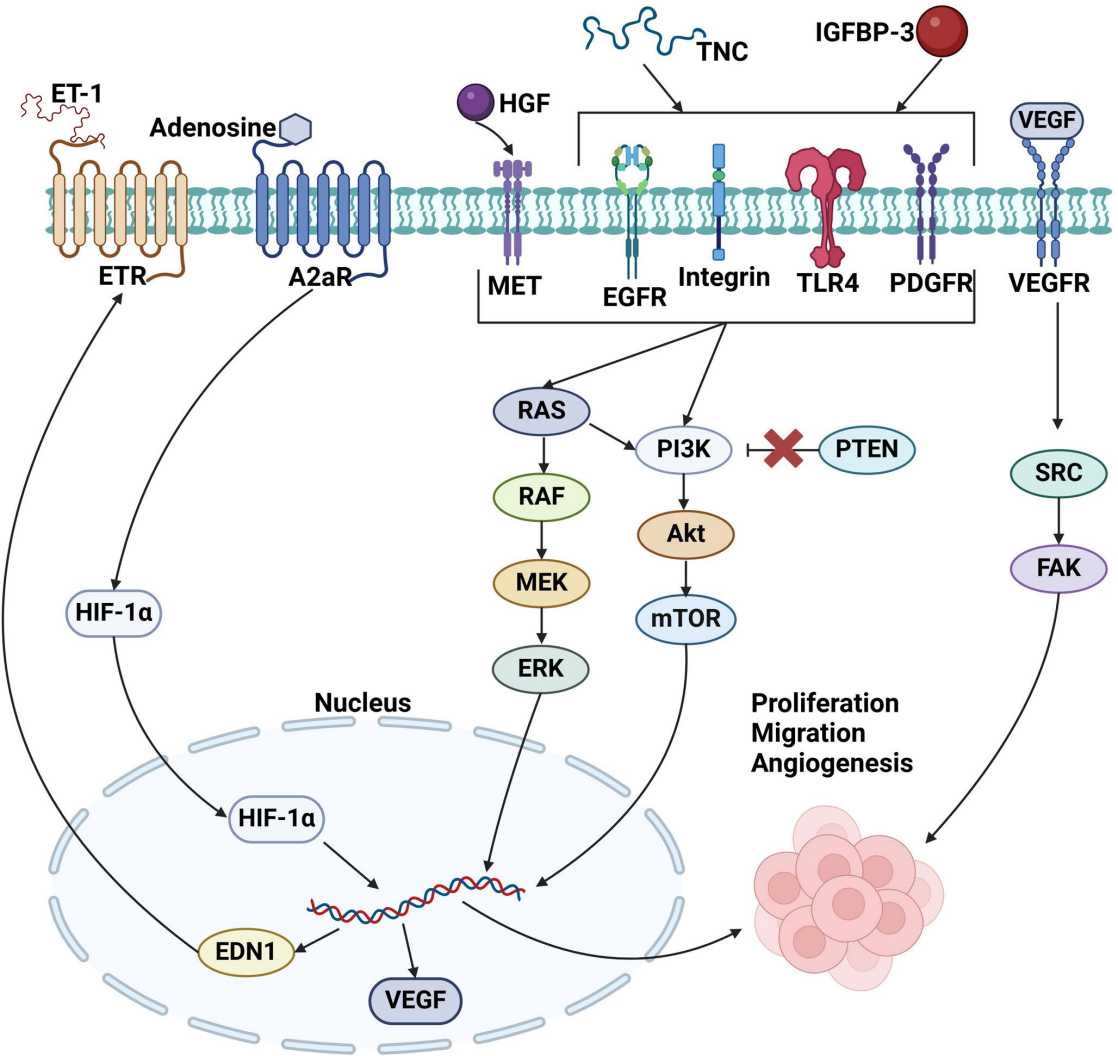
Signaling pathways involved in regenerative functions of CSCs

There are diverse array of native CSCs exhibiting regenerative properties, generally through immunomodulation/healing or angiogenesis. The mechanism of action of these cells is either an internal signaling cascade following ischemia or paracrine activities (Fig. 3). However, some cell types present challenges in isolating them from other endogenous cardiac cells.

**Table 1:** Endogenous CSC sources and their respective genetic/surface markers and functions

Cell type	Genetic/surface marker	Function	References
c-kit + CSCs	c-kit, NKX205, GATA4, and MEF2C	Clonogenicity, induce differentiation of stromal cells into cardiac cells	[17, 20, 23, 24]
Sca-1 + CSCs	Sca-1, GATA4, MEF2C, and TEF1	Vascularization	[29–31]
Cardiosphere-derived cells	GATA4, MEF2C, and MKX2-5	Paracrine activities promoting healing and reduced apoptosis	[33, 35, 39, 40]
Side population cells	NKX2-5, MEF2C, GATA4, and Abcg2	Cell growth and proliferation, differentiation into cardiac cells	[18, 21, 39, 40]
Bmi-1 + CSCs	Bmi-1, NKX205, GATA4, and MEF2C	Cell growth and proliferation, differentiation into cardiac cells	[22, 44–46]
Isl-1 + CSCs	Isl-1, NKX205, GATA4, and MEF2C	CM growth during early developmental stage	[18, 75, 76]
Cardiac atrial appendage cells	NKX205, GATA4, and MEF2C	Secrete aldehyde dehydrogenase to protect against oxidative stress and secrete angiogenic factors	[10, 20, 50]
Cardiac adipose-derived cells	MEF2C, cTnT, NKX2-5, TBX5, IRX4	Differentiation into CM, secretion of factors that protect against apoptosis, inflammation, and fibrosis	[35, 58–60, 63]
Epicardial cells	NKX205, GATA4, and MEF2C	Paracrine activities promoting healing and reduced apoptosis	[21, 72]

### Evidence from translational models

The dogmatic nature of ischemic cardiac damage being permanent has become increasingly problematic as rates of cardiovascular disease continue to climb, accounting for roughly 17.9 million deaths annually, as reported by the World Health Organization in 2019 [21]. Not until 2009 was this belief refuted, as Bergmann et al. [79] determined that the Heart can self-renew up to 50% of its CM within an adult lifespan [79]. Hsieh et al. [80] showed that up to 15% of CM regenerate following ischemic injury from non-CM cells and possibly stromal cells [80]. Since these groundbreaking discoveries, emerging research has demonstrated the immense therapeutic potential of hPSCs in regenerating various cell lineages throughout the body. Unfortunately, hPSCs present challenges regarding their regenerative potential following ischemic damage [5, 6]. Specific challenges include isolating and differentiating hPSCs into the appropriate cardiac lineage [8]. These considerations have led to investigating the therapeutic potential and regenerative capacity of resident CPCs, of which various subtypes listed above have been isolated and tested (Fig. 2**and** Table 1).

Additionally, exploration into CPCs and their subtypes has been proven advantageous over hPSCs in terms of sustainability post-transplantation, adverse outcomes, and cost-effectiveness. The low immunogenicity, stable phenotype, and metabolic stability, decreased tumorigenicity, and increased survival rates of CPCs present a lesser risk for rejection suggesting the translational relevance [81]. Promising solutions to the challenges associated with cardiac stromal cell therapy such as low retention/engraftment are being addressed through repeated dosing, hydrogel carriers, and cardiac patches. Notably, injury targeting has been addressed through magnetic targeting of stromal cells pre-labeled with iron particles; and tumorigenicity/immunogenicity through the alternative cell-free agents including microvesicles and exosomes [82].

C-kit + cells, has been a hot topic of discussion over the last decade. However, an important study by Van Berlo et al. [23] concluded that limited density of c-kit + cells contributed to regenerating ~ 0.03% of CM following cardiac injury; instead contributed more towards endothelial cells and fibroblasts, making them functionally insignificant [23]. Importantly, other researchers proposed that the heart consists of co-existing low KIT and high KIT progenitor cells, with only the latter having regenerative capabilities [83]. Utilization of more sensitive markers and lineage strategies showed c-kit + cells to be prolific in fetal hearts and into adulthood [84, 85]. However, as Van Berlo stated, c-kit + cellular identity and regenerative capacity were endothelial, with rare differentiation into CM following MI challenging their translational potential. Multiple preclinical studies demonstrated c-kit + cells exhibiting myogenic and vessel-forming potential in various animal models to prompt phase 1 clinical trials (SCIPIO). The findings demonstrated reduced infarct size after four months and improved left ventricular contractility after one year in patients following intracoronary KIT + cell delivery [18, 86]. The phase 2 clinical trial (CONCERT-HF) reported treatment with autologous bone marrow-derived mesenchymal stromal cells (MSCs) and c-kit + CPCs is safe and feasible [24].

The paracrine effects of c-kit + cells, different methods of transporting such therapies, and possible explanations for variability in regenerative potential have been emerging. C-kit + cells’ paracrine effects have been attributed to angiogenesis rather than generating CM following ischemic injury. This proangiogenic effect has been linked to certain exosomal microRNAs (miR), specifically miR-210-3p and miR-214, within CPCs and their extracellular vesicles (EVs). Multiple studies have found that hypoxia upregulates miRNA-driven paracrine pathways and hypoxic pre-treatment promotes cardiac endothelial cell proliferation, migration, and tube-forming potential while inhibiting apoptosis. Conversely, silencing or inhibiting miR-210-3p/miR-214 expression after hypoxia treatment suppressed the secretion of proteins that promote angiogenesis [87, 88]. Similarly, pretreatment of c-kit + cells with bradykinin, enhanced exosomal miR-3059-5p mediated angiogenesis and cardioprotection [89]. C-kit + progenitor cells seeded on human-derived decellularized extracellular matrix scaffolds demonstrated increased stromal cell adhesion and proliferation and, more importantly, differentiation into CM-like cells after 14 days of culturing without any inducing factors necessary [90, 91] These findings supported the translational possibilities of pretreatment for successful clinical outcomes.

The cell cycle-modulating properties of CSCs encourage proliferation of healthy CM following ischemic injury. cSPCs and Bmi1 + cells contain surface markers that facilitate cell-cycle entry, suggesting that reprogramming these cells activates the proliferation and growth of CM. Dual therapy of CSCs in conjunction with other stromal cell types is a promising alternative. A study by Avolio et al. [92] reported the improved healing of infarcted cardiac tissue following combination treatment with CSCs and saphenous vein-derived pericytes (SVPs) [92]. In isolation, these cells resulted in enhanced contractility, increased revascularization, endogenous stromal cell recruitment, and CM proliferation. Similarly, Park et al. [93] showed amplified cardiac repair following treatment with a human mesenchymal stomal cell (hMSC) patch and intramyocardially injection of hPSC-CM. This combination resulted in prolonged paracrine signaling and improved function of the injected CM [93]. Bargehr et al. [94] showed that human embryonic stromal cell (hESC)-derived epicardium co-transplanted with CM improved vascularization and enhanced graft size [94]. Table 2 demonstrates the major pre-clinical outcomes and translational potential of CSCs. These findings suggest a translational future for endogenous CSCs in combination with other stromal cell types.

**Table 2:** Endogenous cardiac cell types and outcomes of studies

Cell type	In vivo/ Ex vivo	Animal model	Outcome	References
C-Kit+	In vitro/in vivo	Rats/Mice	Exhibit regenerative and clonogenic properties with affinity for differentiation into CM, endothelial cells, and smooth muscle cells with role in expansion of CM post infarction	[18, 22]
Sca-1+	In vivo	Mice	Plays a role in healing process due to hematopoietic properties and angiogenesis rather than proliferation and growth of new CM. Some studies have shown myogenic potential	[22, 30, 31]
Cardiosphere- derived	In vivo/In vitro	Rats, Mouse, Pigs	Injection led to CM, vascular, and endothelial cell proliferation. Exosomes from cells exhibit paracrine effects encouraging angiogenesis, proliferation, and improved CM survival with scar reduction, increased viable myocardium, and enhanced function post injury	[22, 33]
Side population	In vivo/In vitro	Mice	Proliferation of CM, endothelial cells, and smooth muscle cells with some ability to differentiate into CM	[18, 39]
Bmi1+	In vivo/In vitro	Mice	Differentiates into CM-like cells, vascular endothelial cells, smooth muscle-like cells. Post-MI, cells mobilize to support revascularization and remodeling via angiogenesis and CM	[44–46]
Isl-1+	In vitro	Mice	Exhibit limited cell expansion due to being small in number, playing limited role in post-injury with little to no involvement in infarct regions and preference to differentiate into endothelial cells. Also noted increased paracrine activity and cell protection via IGFBP-3 pathway	[18, 76]
Cardiac Atrial Appendage	Ex vivo/In vitro	Human	Exhibit high aldehyde dehydrogenase activity, protecting these cells against cytotoxicity and ischemia with ability to secrete angiogenic factors	[20, 51]
Cardiac Adipose	In vivo	Mice	Ability to produce beating phenotype CM and also secrete factors that protect myocardial cells from apoptosis, inflammation, and fibrosis	[60–62]
Epicardial	In vivo	Mice	Differentiate mainly into SMCs/fibroblasts and secrete paracrine factors, which support angiogenesis, protect the myocardium, and recover cardiac function	[72]

### Challenges and controversies in cardiac stem cell therapy

Despite the encouraging preclinical findings with cardiac stromal cells, the broader field of cardiac cell therapy has faced persistent challenges and controversy. Nigro et al. highlighted that following more than 15 years of clinical trials, stem cell therapy has largely failed to deliver consistent improvements in patient outcomes, citing variable trial designs, modest efficacy, and difficulties in reproducibility [95]. Similarly, Bolli and Tang described the “sad plight” of cardiac cell therapy, emphasizing poor cell survival, limited engraftment, and heterogeneous study endpoints as factors that have prevented the translation of promising laboratory findings into meaningful clinical success [96]. A recent update by Zhang et al. underscored that despite hundreds of trials worldwide, most studies continue to show modest, if any, benefit in reducing heart failure progression, and no trial has yet demonstrated a reproducible improvement in clinical outcomes such as mortality [97]. Further, the ESC Heart Failure task force (Povsic et al.) has called for a critical reassessment of the field, noting issues of trial integrity, publication bias, and the need for standardization in outcome measures and patient selection [98]. Together, these critiques highlight that while endogenous CSCs remain biologically intriguing and potentially advantageous over extra-cardiac cell types, the larger body of work in cell therapy for heart disease has not yet been delivered on its initial promise. This warrants the importance of cautious interpretation, rigorous trial design, and further mechanistic studies prior to CSC-based therapies and clinical interventions.

## Conclusion

The current evidence regarding CSCs for regenerative therapy suggests that they may play an essential role in the healing and recruitment process rather than producing new CM. Diverse endogenous CSCs demonstrate paracrine activities, immunomodulation, cell cycle modulation, and/or the ability to differentiate into endothelial cells to aid in angiogenesis post-injury. Future efforts with CSC research are warranting to extrapolate CSCs into the clinical arena of cardiac healing and regeneration.

## Data Availability

No datasets were generated or analysed during the current study.

## Abbreviations

ADSC: Adipose derived stromal cell
AGE: Advanced glycation end-product
BM-HSC: Hematopoietic bone marrow stromal cell
BMI1: B cell-specific Moloney murine leukemia virus integration site 1
BMMNC: Bone marrow-derived mononuclear cell
CASC: Cardiac atrial appendage stromal cell
CM: Cardiomyocyte
CSC: Cardiac stromal cell
CPC: Cardiac progenitor cell
cSPC: Cardiac side population cell
EpC: Epicardial cells
hMSC: Human mesenchymal stromal cell
hPSC: Human pluripotent stromal cell
IGFBP-3: Insulin-like growth factor binding protein
MI: Myocardial infarction
MSC: Mesenchymal stromal cell
